# Beneficial Effect of TRAIL on HIV Burden, without Detectable Immune Consequences

**DOI:** 10.1371/journal.pone.0003096

**Published:** 2008-08-28

**Authors:** Brett D. Shepard, Davide De Forni, David R. McNamara, Andrea Foli, Stacey A. Rizza, Roshini S. Abraham, Keith Knutson, Peter J. Wettstein, Franco Lori, Andrew D. Badley

**Affiliations:** 1 Mayo Clinic, Rochester, Minnesota, United States of America; 2 Virostatics s. r. l., Sassari, Italy; 3 Fondazione Istituto di Ricovero e Cura a Carattere Scientifico Policlinico San Matteo, Pavia, Italy; 4 Research Institute for Genetic and Human Therapy, Pavia, Italy; Oregon Health & Science University, United States of America

## Abstract

**Background:**

During uncontrolled HIV disease, both TNF-related apoptosis inducing ligand (TRAIL) and TRAIL receptor expression are increased. Enhanced TRAIL sensitivity is due to TRAIL receptor up-regulation induced by gp120. As a result of successful antiretroviral therapy TRAIL is down-regulated, and there are fewer TRAIL-sensitive cells. In this setting, we hypothesized that all cells that contain virus, including those productively- and latently-infected, have necessarily been “primed” by gp120 and remain TRAIL-sensitive, whereas uninfected cells remain relatively TRAIL-resistant.

**Methods and Findings:**

We evaluated the immunologic and antiviral effects of TRAIL in peripheral blood lymphocytes collected from HIV-infected patients with suppressed viral replication. The peripheral blood lymphocytes were treated with recombinant TRAIL or an equivalent amount of bovine serum albumin as a negative control. Treated cells were then analyzed by quantitative flow cytometry, ELISPOT for CD4+ and CD8+ T-cell function, and limiting dilution microculture for viral burden. Alterations in the cytokine milieu of treated cells were assessed with a multiplex cytokine assay. Treatment with recombinant TRAIL *in vitro* reduced viral burden in lymphocytes collected from HIV-infected patients with suppressed viral load. TRAIL treatment did not alter the cytokine milieu of treated cells. Moreover, treatment with recombinant TRAIL had no adverse effect on either the quantity or function of immune cells from HIV-infected patients with suppressed viral replication.

**Conclusions:**

TRAIL treatment may be an important adjunct to antiretroviral therapy, even in patients with suppressed viral replication, perhaps by inducing apoptosis in cells with latent HIV reservoirs. The absence of adverse effect on the quantity or function of immune cells from HIV-infected patients suggests that there is not a significant level of “bystander death” in uninfected cells.

## Introduction

The role of tumor necrosis factor-related apoptosis inducing ligand (TRAIL) during HIV infection remains controversial. Exploitation of TRAIL signaling and subsequent TRAIL-induced apoptosis may have therapeutic value by promoting the death of cells which harbor latent HIV reservoirs in HIV-infected patients. Consistent with this hypothesis, leucine-zipper TRAIL and agonistic anti-TRAIL receptor antibodies [1] or autologous activated NK cells expressing TRAIL [2] induced *in vitro* apoptosis of monocyte-derived macrophages and peripheral blood lymphocytes from HIV-infected patients, resulting in reduced viral burden following limiting dilution microculture. In all cases tested TRAIL treatment of cells led to reduced viral burden, even to undetectable levels, whereas untreated cells produced large amounts of both HIV RNA and p24 antigen [1], [2]. Furthermore, monocyte-derived macrophages and peripheral blood lymphocytes collected from uninfected donors did not die following treatment with leucine-zipper TRAIL [1]. However, in order for TRAIL therapy to have clinical utility, it must have an acceptable safety profile in HIV patients.

Despite evidence that TRAIL is not toxic to uninfected cells other models have been proposed that suggest TRAIL-induced apoptosis contributes to the “bystander death” of uninfected CD4+ T cells. In these models productive HIV infection enhances TRAIL production, and gp120 “priming” of uninfected T cells induces TRAIL sensitivity. For instance, increased levels of mRNA for IFN-α, TRAIL and TRAIL receptor R2 (TRAIL-R2) were measured in the tonsils of patients with progressive HIV disease compared to those with nonprogressive disease, and tonsils from uninfected patients did not contain TRAIL or TRAIL-R2 mRNA [3]. Because HIV-induced expression of TRAIL is dependent on IFN-α which is primarily produced by plasmacytoid dendritic cells (pDC) [4], a model has been suggested in which HIV induces plasmacytoid dendritic cells to produce INF-α [4]. IFN-α then induces expression of TRAIL in uninfected CD4+ T cells [4]. Subsequent binding of either infectious or noninfectious HIV to uninfected T cells induces expression of TRAIL-R2 [5] leading to apoptosis of the cells and their neighbors via “bystander death” [4].

A second model of TRAIL-mediated death of uninfected cells has been proposed. Massive apoptosis of uninfected CD4+ T cells was observed in the spleens of HIV-infected mice transplanted with human peripheral blood lymphocytes, and the apoptotic cells co-localized with TRAIL-expressing T cells [6]. A subsequent study showed that treatment of uninfected primary human macrophages with HIV-1 Tat protein induced TRAIL expression to levels comparable to those measured following HIV infection [7]. Based on these data a model was proposed in which extracellular Tat produced by HIV-infected cells, induces TRAIL expression in uninfected macrophages [7] and subsequent interaction of these cells with uninfected gp120 “primed” CD4+ T cells results in increased TRAIL sensitivity [5] and consequent TRAIL-mediated apoptosis in uninfected T cells.

However, the definitive experiment using antagonistic anti-TRAIL antibodies has not been tested in these models. In a model of HIV-infected macrophage killing of uninfected T cells anti-Fas antibodies abrogate apoptosis in the uninfected cells [8], [9], yet antagonistic anti-TRAIL antibodies have no such effect (Unpublished results, ADB). Furthermore, in HIV-infected human lymphocyte aggregate cultures containing pDC, antagonistic anti-TRAIL antibodies did not alter uninfected CD4+ T-cell death [10], thereby raising questions as to the true relevance of TRAIL in bystander killing. Moreover, the latter model has been challenged by more recent data demonstrating that HIV Tat increases c-FLIP and downregulates caspase 10 in T lymphocytes [11] and increases Bcl-2 in monocytes [12], each leading to inhibition of TRAIL-mediated apoptosis in each cell type. Lastly, a potential role for TRAIL causing immune cell depletion is further argued against by the recent finding that HIV-infected macrophages upregulate Bfl-1 and Mcl-1 as a counter-regulatory mechanism to resist the cytotoxic effects of TRAIL [13].

Even without such limitations, neither model is mutually exclusive with our previous data showing that uninfected macrophages and lymphocytes do not die following treatment with leucine-zipper TRAIL [1]. Although TRAIL may account for a degree of bystander killing of uninfected CD4+ T cells in patients with unsuppressed replication, it does not preclude a therapeutic role for TRAIL in suppressed patients. Indeed, in suppressed patients with no detectable viral load in the plasma, HIV DNA can be detected in the lymph nodes and peripheral blood lymphocytes (PBLs), reflecting a pool of latently-infected cells established very early during primary HIV infection [14]. While the frequency of these latently-infected cells is approximately 16 per million PBLs [15], thus precluding direct experimentation with this important pool of cells, their persistence despite effective antiretroviral therapy highlights the need for additional strategies of therapy. Since all infected cells, including latently-infected cells which revert to a resting memory phenotype, have necessarily contacted gp120, all such cells would be predicted to have enhanced TRAIL sensitivity. In a suppressed setting lack of replication would predictably reduce TRAIL production, and indeed TRAIL levels are directly proportional to HIV viral load [16], [17]. However, both productively- and latently-infected cells, but very few uninfected cells, should maintain TRAIL sensitivity. Consequently, exogenous TRAIL might selectively target and kill those infected cells. The purpose of the current study was to validate the antiviral properties of TRAIL in suppressed patients and to evaluate whether additional, significant bystander death occurs and deleteriously affects other parts of the immune system.

## Results

**Figure 1:**
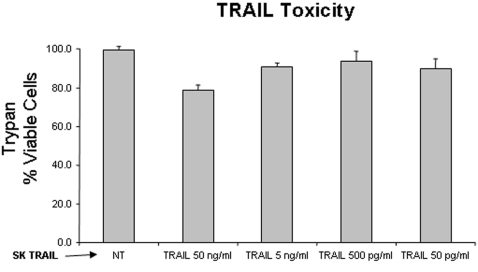
Effect of skTRAIL treatment on cell viability. Peripheral blood lymphocytes (PBLs) were collected from HIV-negative donors, stimulated with PHA and IL-2, and treated with with skTRAIL at the indicated concentrations. Viability was assessed by trypan blue staining.

Human peripheral blood lymphocytes (PBLs) obtained from HIV-negative donors were stimulated with PHA and IL-2 and tested for the cytotoxic response to recombinant TRAIL (skTRAIL) (Fig. 1). A dose of 5 ng/ml of skTRAIL was chosen for subsequent experiments given that this was the maximum dose of skTRAIL that had only limited impact on cell viability.

**Figure 2:**
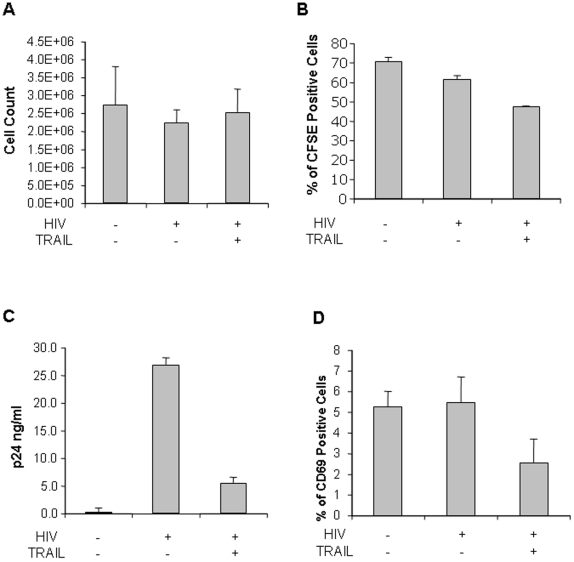
Effects of skTRAIL treatment following *in vitro* HIV infection of PBLs from uninfected donors. PBLs were collected from HIV-negative donors and infected with HIV-IIIB prior to treatment with skTRAIL. The effects on cell count (A), proliferation as assessed by CFSE dilution (B), p24 production (C), and CD69 expression (D) were measured.

PBLs from HIV-uninfected donors were infected with HIV-IIIB and the effect of TRAIL treatment on cell viability was assessed (Fig. 2A). In these experiments, daily TRAIL treatments for ten days had no significant effect on cumulative cell number (Fig. 2A). However, since TRAIL treatment can result in both apoptosis and proliferation, it is unclear whether apoptosis or proliferation occurred in these populations or whether apoptosis occurred early and cell number was rescued by enhanced proliferation. To assess this possibility, cells were stained on day 0 (immediately following HIV infection) with CFSE and proliferation assessed by comparing CFSE dilution between HIV-infected but untreated, and HIV-infected but TRAIL-treated cells (Fig. 2B).

Enhanced cell proliferation might in theory drive HIV replication to such a degree that the antiviral effects of TRAIL treatment might be overcome by enhanced viral replication associated with NF-κB driven proliferation. Consequently, we analyzed whether ten days of treatment with TRAIL maintained the antiviral effect of TRAIL that we have previously observed in short-term treatment studies (Fig. 2C). Indeed, in these studies, HIV-infected but untreated cultures produced significant amounts of p24 that were significantly reduced by treatment with 5 ng/ml of TRAIL on a daily basis. Lastly, treatment decreased the percentage of HIV-infected cells expressing CD69, a cell surface marker of systemic immune activation (Fig. 2D). Altogether, these data suggest that the pro-apoptotic effects of TRAIL on infected cells are sufficient to overcome the proliferative effects of TRAIL in the presumably uninfected T cell populations. Moreover, these data suggest that combining TRAIL treatment with effective antiretroviral therapy might be an efficient way to reduce viral burden.

**Figure 3:**
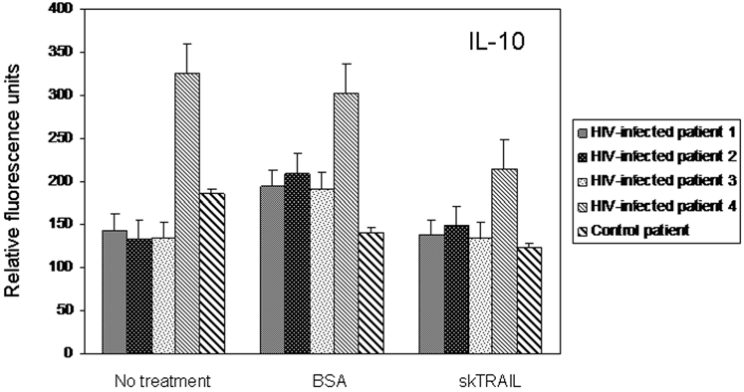
IL-10 expression following treatment of HIV-infected PBLs with skTRAIL, a representative example of results obtained from assays with the Bio-Plex human cytokine 27-plex panel. PBLs were collected from 4 HIV-infected donors and 1 uninfected control. Cells received either no treatment or were treated with either BSA (5 ng/ml) or skTRAIL (5 ng/ml). Following 24 h of culture, supernatants were collected for measurement of cytokine expression. Values are presented as relative fluorescence units, reflecting a range of concentrations from 0.08 to 2.01 pg/ml based on extrapolation from a standard curve generated with known concentrations of IL-10 at the time of analysis.

Because exogenous TRAIL treatment may alter the cytokine milieu of treated cells, multiplex cytokine assays were performed using culture supernatants from cells collected from HIV-infected donors and treated with TRAIL. In comparison to either untreated cells or cells treated with bovine serum albumin (BSA), HIV-infected cells treated with TRAIL showed no biologically significant alterations in cytokine expression. Negligible changes in IL-10 expression were detected following TRAIL treatment of cells collected from 4 HIV-infected patients (Fig. 3). However, the magnitude of change was minimal. For example, IL-10 expression decreased by only 1.37 pg/ml following TRAIL treatment when compared to untreated cells. Similar negligible decreases were detected in IL-8 expression following TRAIL treatment (data not shown). However, we detected no changes in expression among the other cytokines included in the multiplex assay, including: IL-1ra, IL-1β, IL-2, IL-4, IL-5, IL-6, IL-7, IL-9, IL-12 (p70), IL-13, IL-15, IL-17, eotaxin, basic FGF, G-CSF, GM-CSF, IFN-γ, IP-10, MCP-1, MIP-1α, MIP-1β, PDGF-BB, RANTES, INF-α, and VEGF (data not shown). Similar results were measured following TRAIL treatment of PBLs from an uninfected donor (data not shown).

**Figure 4:**
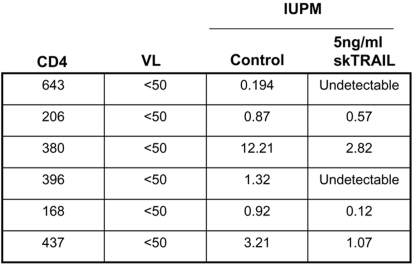
Superkiller TRAIL decreases HIV viral burden in PBLs from HIV infected individuals. PBLs from HIV infected individuals were isolated and treated with skTRAIL or BSA in a limiting dilution micro co-culture. HIV viral burden is reported as infectious units per million.

In order to determine if exogenous TRAIL treatment has beneficial antiviral properties, PBLs were isolated from 6 HIV-infected individuals with suppressed levels of viral replication. All HIV-infected subjects had CD4+ counts greater than 150 cells/mL and had HIV viral loads that were suppressed to <50 (Fig. 4). The PBLs were then treated with 5 ng/mL of TRAIL or equal molar concentrations of BSA and cultured with labeled feeder cells in a limiting dilution microculture. Consistent with prior results [1], [2] and as shown in Figure 4, PBLs from all six individuals had significantly lower infectious units per million (IUPM) when cultured with TRAIL versus those cultured with BSA. PBL cultures from two individuals decreased HIV IUPM to undetectable levels after TRAIL treatment, together demonstrating that exogenous TRAIL therapy *in vitro* decreases HIV viral burden in PBLs from HIV-infected individuals with baseline suppressed HIV viral loads.

**Figure 5:**
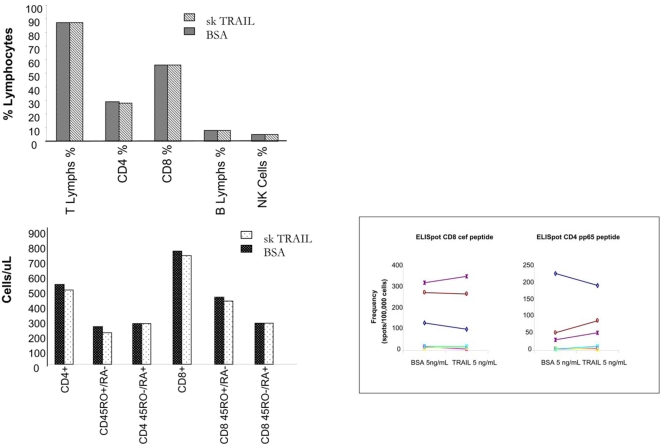
Exogenous superkiller TRAIL does not cause deleterious effects on immune cell numbers or function. PBLs from HIV infected subjects were cultured with skTRAIL or BSA and analyzed for percent of T, B and NK cells (A, top panel), and percent CD4+ and CD8+ memory and naïve T cells (A, bottom panel) by flow cytometry. Cultured PBLs were also analyzed for CD4 and CD8 recall responses by ELISPOT (B).

The antiviral effects of exogenous TRAIL treatment during HIV infection will only be useful if the TRAIL targets and kills HIV-infected cells, but does not affect the remainder of the immune system. Therefore, PBLs from the 6 HIV-infected individuals were cultured with 5 ng/mL of TRAIL or BSA to determine if TRAIL altered T cell, B cell, or NK cells numbers, or CD4+ and CD8+ T-cell function. Figure 5A demonstrates that TRAIL treatment did not alter the percent of total T lymphocytes, CD4+ or CD8+ T cells, B cell or NK cell percentages, as determined by flow cytometry (top panel). Flow cytometry for the surface markers CD45 RO+/RA− and CD45RO−/RA− demonstrated that TRAIL treatment did not alter CD4+ or CD8+ memory or naïve populations (Fig. 5A, bottom panel). Furthermore, ELISPOT analysis showed no difference between CD4+ recall responses to the CMV pp65 peptide or the CD8+ CEF peptide in cells cultured with TRAIL or control protein (Fig. 5B). Thus, suggesting that exogenous TRAIL treatment effectively decreases HIV viral burden in PBLs from HIV-infected individuals and has no adverse effects on immune cell numbers or function.

## Discussion

The present study shows that recombinant TRAIL can reduce HIV viral burden, even in lymphocytes collected from HIV-infected patients with a suppressed viral load, suggesting that TRAIL treatment may be an important adjunct to antiretroviral therapy, perhaps by inducing apoptosis in cells with latent HIV reservoirs.

TRAIL treatment did not alter the cytokine milieu of treated cells. Of equal significance, the present study shows that treatment with recombinant TRAIL had no adverse effect on either the quantity or function of immune cells from HIV-infected patients with suppressed viral replication, which suggests that there is not a significant level of “bystander death” in uninfected cells from these suppressed patients. These data challenge suggested models to the contrary, which have not yet been definitively tested with antagonistic anti-TRAIL antibodies to show that TRAIL inhibition abrogates apoptosis in uninfected cells. The results of these quantitative and functional assays in lymphocytes from infected patients supplement an earlier observation that treatment with recombinant TRAIL did not induce apoptosis in peripheral blood lymphocytes collected from uninfected donors [1]. Furthermore, these results are consistent with a growing body of preclinical and clinical trial data which have not shown significant toxicity with use of recombinant, untagged TRAIL in HIV-negative cancer patients [18]. A prior study raised concern after showing that treatment with polyhistidine-tagged TRAIL was toxic to primary human hepatocytes (PHH) [19]. However, it is now widely accepted that the toxicity was due to formation of highly oligomerized aggregates of this tagged version of TRAIL, which are toxic to normal tissues through enhanced multimerization of TRAIL receptors [20]. Indeed, subsequent studies have shown that three tagged versions of TRAIL were toxic to PHH, whereas untagged TRAIL was not [21]. Moreover, we have observed minimal toxicity of leucine-zipper TRAIL to freshly isolated PHH when used at the doses in the current report (Personal observation, ADB and SAR).

The mechanism by which activation of TRAIL signaling induces reductions in HIV viral load is uncertain. TRAIL-mediated apoptosis may play a role in the cytolytic clearance of virus-infected cells, and the gene encoding TRAIL is one of the earliest induced by interferons in response to viral infection [22]. Alternatively, induction of TRAIL signaling may promote reductions in HIV viral load through increased immune activation. Indeed, TRAIL signaling has been shown to induce non-apoptotic pathways through the activation of NF-κB, PKB/Akt, and MAPK [23]. However, our data demonstrating decreased CD69 expression following TRAIL treatment of PBLs infected *in vitro* with HIV raises uncertainty regarding this hypothesis. Because of the uncertainty of the mechanism that links induction of TRAIL signaling with subsequent reductions in HIV viral load and the potential therapeutic value that may be gained by such an understanding, the use of TRAIL in the treatment of HIV infection is an obvious and logical candidate for further study.

## Materials and Methods

### Patient consent and protocol

Patient samples were obtained following informed consent, the protocol of which was reviewed and approved by the Mayo Clinic institutional review board.

### TRAIL

SuperKillerTRAIL (Axxora Life Sciences, Inc.) and bovine serum albumin (Sigma) were each diluted in phosphate-buffered saline to a working stock of 100 ng/µl prior to use in experiments

### Cells

Human peripheral blood lymphocytes (PBLs), obtained from healthy, normal donors, were stimulated with PHA 5 µg/ml. Then IL-2 was added (20 U/ml) and cells were plated (10^5^ cells per well in 200 µl) for testing of the cytotoxic response to treatment with TRAIL. Unstimulated PBLs from healthy, normal donors were used for *in vitro* HIV infection as described below. Unstimulated PBLs from 6 HIV-infected patients with suppressed viral replication were used in experiments to determine if exogenous TRAIL treatment has beneficial antiviral effects. All cells were isolated from whole blood samples and were enriched for PBLs using Ficoll-Paque (GE Healthcare, Ltd.) density gradient centrifugation according to the manufacturer's recommendations.

### HIV infection of PBLs from healthy donors

Unstimulated (quiescent/resting, day 0–5) human CD4+ T-lymphocytes, obtained by magnetic beads separation from healthy, normal donors, were infected with HIV-1 IIIB, defective for the regulatory genes *vpr* and *nef*. After five days in culture cells were stimulated with PHA (5 µg/ml), then at day 7 IL-2 (20 U/ml) was added (stimulated, day 6–10). A 5 ng/ml concentration of TRAIL was used in the experiments. At day 10 cells and supernatants were harvested and HIV-1 p24 antigen was measured by ELISA. Viability of cells was investigated at day 10 by trypan blue staining. Proliferation of CD4+ T cells was studied by staining the cells with carboxyfluorescein diacetate-succinimidyl ester (CFSE) at day 0 (immediately after infection). Activation was evaluated by studying the expression of CD69.

### Multiplex cytokine assays

CD4+ T lymphocytes were isolated from whole blood samples collected from 4 HIV-infected patients with suppressed viral replication and 1 healthy, uninfected control subject. Enrichment for CD4+ cells was achieved through the use of the RosetteSep human CD4+ T cell enrichment cocktail (StemCell Technologies) according to the manufacturer's protocol. Following isolation, 5×10^6^ CD4+ T lymphocytes from each subject were resuspended in 500 µl of fresh RPMI media and incubated at physiologic conditions for 24 hours without treatment, or with either bovine serum albumin (5 ng/ml) or TRAIL (5 ng/ml). Following incubation, supernatant from each experiment was collected and residual cells were removed by centrifugation. Supernatants were then used in muliplex cytokine assays using the Bio-Plex Human Cytokine 27-plex Panel (Bio-Rad Laboratories) according to the manufacturer's instructions. Samples were analyzed on a Luminex suspension array system (Bio-Rad Laboratories) and subsequently quantitated using Bio-Plex Manager software. (Bio-Rad Laboratories), each according to the manufacturer's instructions.

### ELISPOT assays

Peripheral blood lymphocytes were isolated from 6 HIV-infected patients with suppressed viral replication and treated with 5 ng/mL of TRAIL or an equivalent amount of BSA. Frequencies of antigen-specific CD4+ and CD8+ T cells were estimated by primary IFN-γ ELISPOT assays. Briefly, CD4+ and CD8+ T cells were enriched from peripheral blood lymphocytes by sequential positive selections with anti-CD4 and anti-CD8 antibody-coated magnetic particles (Miltenyi Biotec). The purity of fractions was routinely monitored and was consistently greater than 90%. CD4+ and CD8+ T cells were stimulated with autologous antigen-presenting cells (non-T cell) pulsed with CMV pp65 peptide and the CMV CEF Control Peptide Pool, respectively. The responder∶stimulator combinations were cultured in microtiter plates coated with anti-IFN-γ antibody (Mabtech) for two days after which the cells were washed out and replaced with biotinylated anti-IFNγ antibody (Mabtech). Streptavidin-conjugated horseradish peroxidase (Vector Laboratories) was added followed by AEC substrate for spot development.

### Flow cytometry

Peripheral blood lymphocytes were isolated from 6 HIV-infected patients with suppressed viral replication and treated with 5 ng/mL of TRAIL or an equivalent amount of BSA. CD3+, CD4+, and CD8+ T cells, CD19+ B-cells and NK (CD16+56+) were quantitated using a 6-color flow cytometric assay with specific monoclonal antibodies for each marker. The assay utilized an FDA-approved panel of reagents (BD Multitest, BD BioSciences) on the BD FACS Canto flow cytometer (BD BioSciences). The assay was performed using 1 ml whole blood collected in an EDTA tube in a 6-color Lyse-no wash procedure. Both the percent of cells in each subpopulation as well as the absolute counts per µl of blood was reported for CD45+ lymphocytes, and the T-cell, B-cell and NK-cell populations. The absolute counts were determined using internal bead standards and data analysis was performed with the BD FACS Canto clinical software (BD BioSciences). Manual gating of populations was performed where needed if the software-assigned gate was deemed to be inaccurate.

### Limiting dilution microculture assays

Viral burden was estimated using limiting dilution microculture assays, followed by maximum likelihood estimates as previously described [24]. We used the maximum available number of cells in each co-culture for each patient. The starting cell concentrations ranged from 3.4×10^6^ to 5.0×10^7^ cells. Following overnight TRAIL or BSA treatment, cells were washed three times and then co-incubated with irradiated feeder cells for 14 days. On day 14, p24 and viral RNA levels were measured. p24 antigen was measured in duplicate using a commercial ELISA assay (NEN, Life Sciences Products). In independent experiments we determined that TRAIL treatment does not impair the ability of resting T cells to become activated (data not shown).
